# Apparatus for Paralysis of the Radial Nerve

**Published:** 1919-01

**Authors:** 


***An Apparatus for Paralysis of the Radial Nerve.*** By Dr. Ripert. *Revue Interalliée pour les Mutilés de la Guerre,* April, 1918.

Paralysis of the radial nerve is very common among wounded soldiers. Apparatus of many sorts have been proposed for this case. Dr. Ripert has contrived a kind to be used in the case of a definitive paralysis of the radial nerve.

It consists of a light fore-arm piece, on the wrist of which is articulated a specially designed metal attachment, maintained by a steel spring, the purpose of which is to support the hand. This attachment can describe part of a circle, and can be fixed at any desired point on this circle, or left free if requested, or even taken off. Both the thumb and the wrist can move freely, and many kinds of work may be performed with the help of this apparatus.

